# Palynological Age Control and Paleoenvironments of the Paleogene Strata in Eastern Dahomey Basin, Southwestern Nigeria

**DOI:** 10.1038/s41598-020-65462-7

**Published:** 2020-06-02

**Authors:** Taiwo A. Bolaji, Otobong S. Ndukwe, Ajibola R. Oyebamiji, Okechukwu N. Ikegwuonu

**Affiliations:** 1 Department of Geology, Federal University Oye-Ekiti, Oye-Ekiti, Nigeria; 2 Department of Geology, Chukwuemeka Odumegwu Ojukwu University, Uli, Nigeria

**Keywords:** Palaeontology, Energy science and technology

## Abstract

Paleogene deposits are extensively exposed in the Eastern Dahomey (Benin) sedimentary Basin in southwestern Nigeria. Outcrop logging and sampling of lithological sections were systematically carried out at the Ibese Quarry, in order to re-establish the age of sediments and reconstruct their depositional environment using samples rich in organic-walled microfossils. Two formations were recognized; the Ewekoro and Akinbo Formations. Two main lithological units were identified; limestones and carbonaceous shales. Minor lithologies include ferrugineous sandstones and glauconite. Results from the palynological examination show that terrestrial palynomorphs (spores and pollen) dominate over the marine dinoflagellates cyst species in the samples recovered from the Ewekoro Formation, while samples from the Akinbo Formation recorded high abundance with less diversity of the marine dinoflagellates cysts over the terrestrial sporomorph. Age determination/correlation was achieved based on selected stratigraphic index taxa recovered. The samples from Ewekoro Formation were dated as late Early Paleocene to early Middle Paleocene, based on the presence of the following pollen key-taxa: *Proxapertites operculatus, Retidiporites magdalenensis, Spinizonocolpites baculatus, Mauritidiites crassiexinus, Scabratriporites simpliformis*, and *Echitriporites trianguliformis*. The samples from the Akinbo Formation were assigned late Middle Paleocene to late Paleocene, based on the presence of the following pollen key-taxa: *Proxapertites operculatus/cursus, Grimsdalea polygonalis, Retibrevitricolpites triangulatus, Psilatricolporites operculatus, Retistephanocolpites williamsi, Bombacidites sp., Apectodinium homomorphum*, and *Apectodinium quinquelatum*. The environmentally significant palynomorph species indicated differences in paleodepositional environments, ranging from shallow marine in the Ewekoro Formation at the base, up the stratigraphic sequence, to marginal marine (estuarine) depositional environment in the overlying Akinbo Formation.

## Introduction

**Figure 1:**
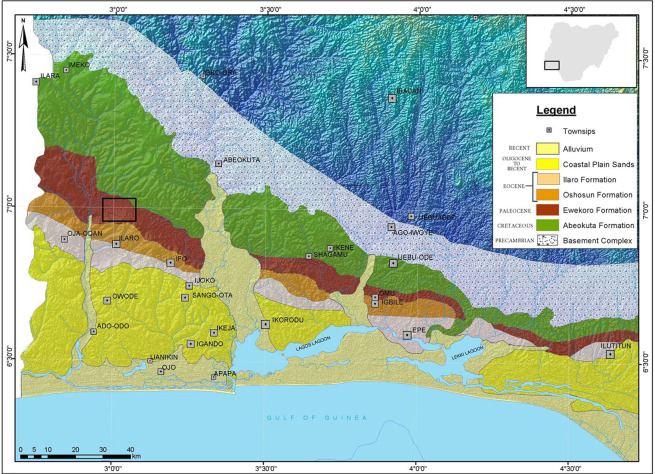
The Geological Map of Eastern Dahomey Basin showing the study area.

The Dahomey Basin is one of the numerous marginal basins formed along the coast of Africa and Brazil following the opening of the South Atlantic. It is an extensive coastal basin located on the margin of the Gulf of Guinea (Fig. 1). The basin is regarded as one of the active basins among the Nigerian sedimentary basins since its subaerial parts are eroding while deposition is taking place in its distal submarine parts. The Dahomey Basin is a combination of the inland/coastal/offshore basin^1^, that stretches from southeastern Ghana through Togo and Republic of Benin to the southwestern part of Nigeria. It was described by^1^ as miogeosyncline, a basin in which volcanism has not been associated with sedimentation. The Eastern Dahomey Basin (the Nigerian sector) comprises of the border flanked by the Benin Republic and Nigeria, and the Benin Hinge line^1^, and consists of approximately 3 km thick Cretaceous to Recent sediments^2^. The basin was further classified into three viz: pre-lower Cretaceous folded, Cretaceous and Tertiary chronological stratigraphic units^2^.

## Previous work

The geological studies of the Cenozoic sediments in the Eastern Dahomey (Benin) Basin have been undertaken by various researchers, with emphasis mostly on the sedimentology, stratigraphical, paleontology, petroleum, and geochemistry^3–7^. The palynological study of the Cenozoic sediments, on the contrary, has received less attention. The few palynological works include^8–14^.

A Paleocene age was assigned to the Kerri-Kerri Formation in Northern Nigeria^8^, based on the presence of the following pollen key-taxa: *Longapertites inornatus, L. marginatus, L. microfoveolatus, Retimonocolpites noremi, Foveomonocolpites bauchiensis, Monocolpites marginatus, Cranwellipollis gombeensis, and Bacutricolpites portiskumensis, Mauriitides crassibaculatus*, and *Proxapertites operculatus*. The Upper Senonian and Lower Tertiary deposits from southern Nigeria were studied by^14^ and Paleocene-Eocene age assigned for the Lower Tertiary strata, based on the presence of the following pollen key-taxa: *Longapertites vanendeenburgi, L. marginatus, Retidiporites magdalenensis, Retistephanocolpites williamsi, Arecipites microreticulatus, Ericipites pachyexinus, Bacutriporites orluensis, E. trianguliformis*. ^13^examined the core samples of the sections of Imo Shale from the Gbekebo-1 well in the Benin Flank of the Niger Delta, and assigned Late Paleocene-Early Eocene age for the samples based on the presence of *Glaphyrocysta exuberans, G.ordinata, Hafniasphaera septata, Hystichokolpoma rigaudiae, Achomosphaera ramulifera, Cordosphaeridium multispinosum, Apectodinum quinquelatum, Cleistosphaeridium spinulastrum, Emmetrocysta urnaformis, Fibrocyata vectense, Wetziella articulata, Adnatosphaeridium vittatum, Impletosphaeridium cracens, I. ligospinosum, Muratodinum fimbritaum, Operculodinium bellunum, Paleocystidinium golzowense, Riculacacysta perforate*, and *Proxapertites operculatus*.

Paleocene-middle Eocene age was assigned to the Paleogene strata of the Afikpo Sub-basin from southeastern Nigeria, using the recovered diagnostic palynomorph-rich samples^11^. They inferred a marginal/shallow marine estuarine depositional condition for the Imo Formation. Late Paleocene (Thanetian) to Early Eocene (Ypresian) age was assigned to the Oshosun Formation in the Eastern Dahomey Basin^9^, using the recovered dinoflagellate cysts: *Apectodinium homomorphum, A. quinquelatum, A. paniculatum, Kallosphaeridum cf. brevibarbatum, Hafniasphaera septate*, and *Ifecysta pachyderma*. They remarked that the formation was deposited in a marginal marine and/or brackish water-estuarine setting. The present study attempts to establish the palynological age and paleoenvironments of the Ewekoro and Akinbo Formations within the Cenozoic succession in the Eastern Dahomey (Benin) Basin, using the organic-walled microfossils rich samples.

## General geology and stratigraphy

**Figure 2:**
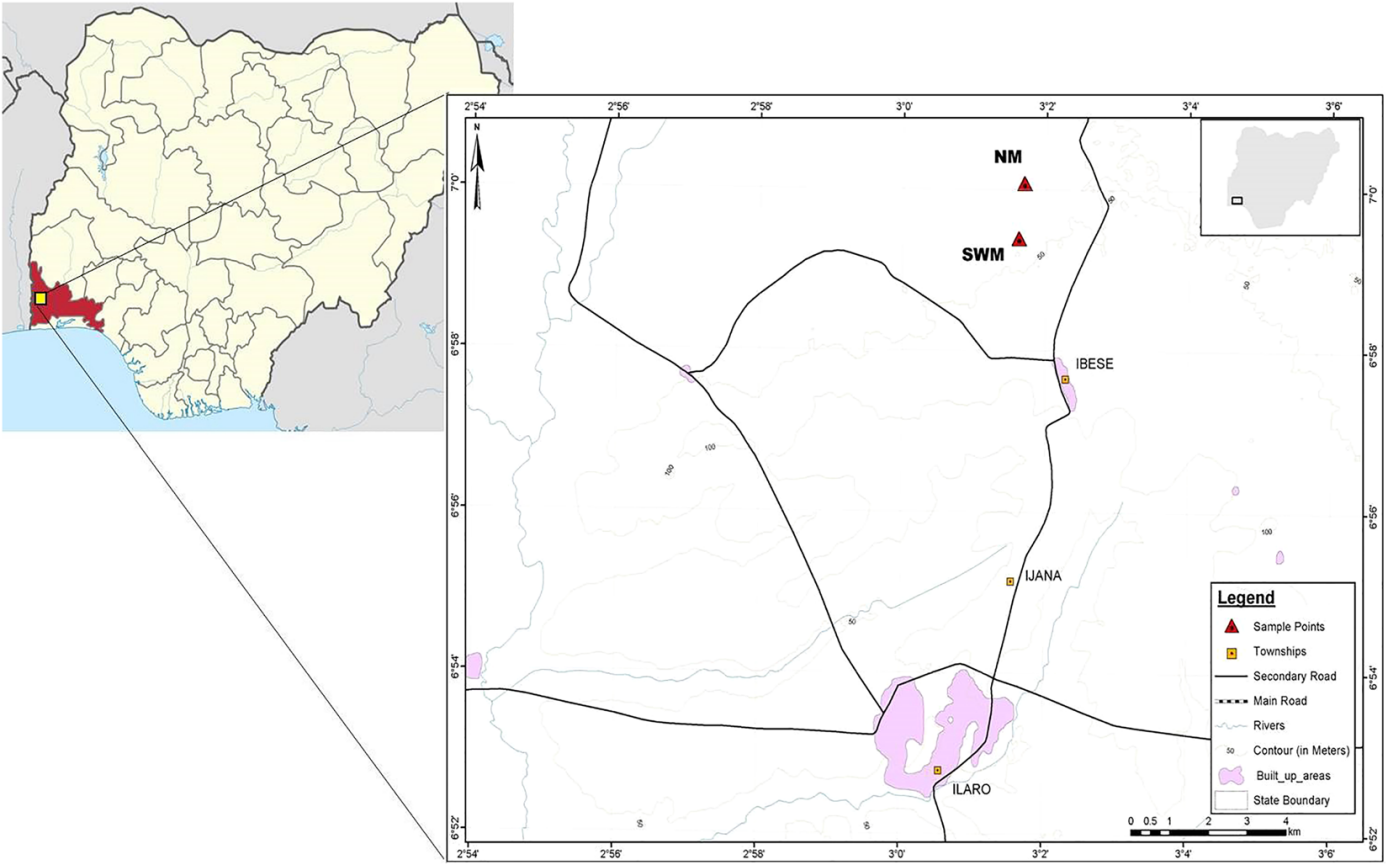
Map of Nigeria showing the location of the study area and sampling points.

The study area (Fig. 2) lies within the eastern part of the Dahomey Basin, which extends eastward into western Nigeria, as far east and west of the Ilesha Spur and the Volta Delta Complex respectively, in Ghana. The basin development began *ca*. 95 million years BP, which led to the initial deposition of the continentally derived basal conglomerate and grits, non-conformably on the Precambrian Basement Complex. The succession, however, has not experienced folding, and the beds are gently dipping S – SSW at *ca*. 1° in accordance with the basement configuration (Fig. 1).

**Figure 3:**
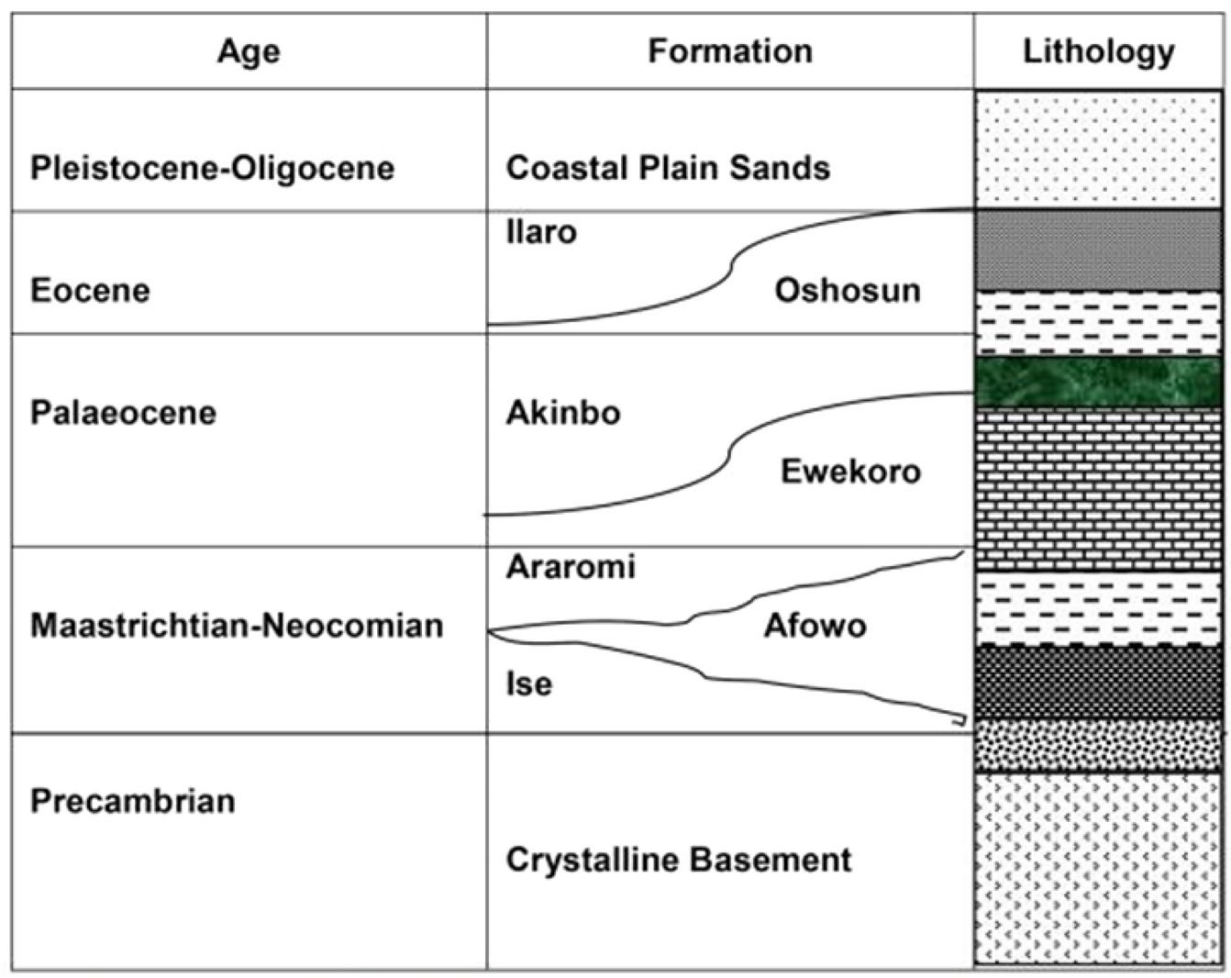
Stratigraphy of Nigerian Eastern Sector of Dahomey Basin (*modified after* Omatsola and Adegoke, 1981).

Several works, including^15–18^, have discussed the stratigraphy of Dahomey Basin. The basin consists of Late Cretaceous to Cenozoic sediments of about 2100 m thick, but thickens markedly into the offshore and thins beneath the deep-water area. The Nigerian sector of the Benin Basin thins into the Basement Complex of the Okitipupa ridge. According to^19^, the basin was cut off from the eastern side, *i.e*. the Anambra Basin and the Niger Delta Basin by a ridge of the crystalline basement (the Okitipupa ridge). The Dahomey Basin fill can broadly be classified into two: the Cretaceous Abeokuta Group, which comprises of the Ise, Afowo, and Araromi; and the Cenozoic units consisting of Ewekoro, Akinbo, Oshosun and Ilaro Formations^17,20^ (Fig. 3).

The Ewekoro Formation is traceable over a distance of 320 km continuously from east of Accra, eastward through the whole of the Dahomey Basin^21^. It is a WNW-ESE trending unit, and consists of light brown to grey arenaceous limestone, with fragmentary bioclast and terrigenous material increasing towards the base^22^. Overlying the Ewekoro Formation is the Akinbo Formation, which consists of shale and clay, and a basal glauconitic band, which forms a prominent horizon that separates it from the topmost unit of the underlying Ewekoro Formation^20^. The Akinbo Formation is overlain by Oshosun Formation, which consists of phosphate-rich, green-grey clay and shale with sandstone interbeds^7^. Overlying the Oshosun Formation are the Ilaro and Benin Formations, which are characterized predominantly by coarse-grained sandstone; and sandy beds of estuarine, deltaic and continental origins.

## Description of stratigraphic sections

**Figure 4:**
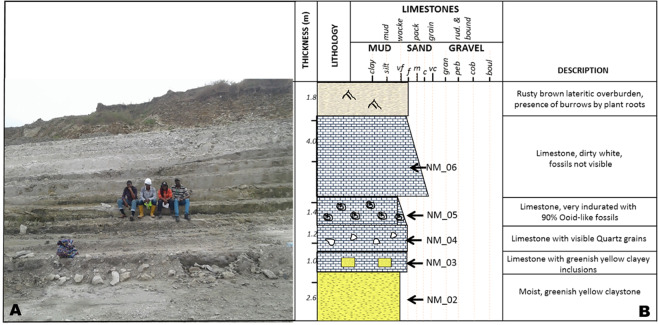
(**A**) Photograph of the sampled section in the Ibese Quarry with team members as scale. (**B**) Lithological log and description of the sampled section with sampled beds marked.

**Figure 5:**
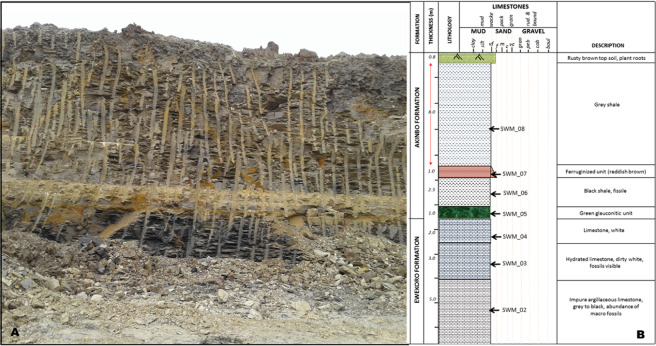
(**A**) Photograph of the sampled section [Height of the quarry wall is 23.1 m]. (**B**) Lithological log and description of the sampled section with the sampled beds marked.

The study area is delimited by latitudes 06°59.440′N and 07°00.75′N, and longitudes 003°01.614′E and 003°01.692′E. It lies within the Cenozoic sequence in the Eastern Dahomey (Benin) Basin, southwestern Nigeria (Figs. 1 and 2). Two formations (Ewekoro and Akinbo), were exposed in the studied area. The sections (Figs. 4A,B and 5A,B) exposed at the Ibese Quarry comprise of two main lithological units, which include limestones and carbonaceous fissile shales, interbedded with a glauconitic band and ferruginized sandstones. The outcrop logging and sampling were systematically carried out from base to top. A total of 12 field samples, distributed as follow, were collected and studied: Ewekoro Formation (8 samples) and Akinbo Formation (4 samples) (Fig. 2). The sections are described as follows:

### Unit 1: Northern Mine (NM)

This section is exposed in the Northern Mine (NM) at the Ibese Quarry (Fig. 4A), with coordinates [003°01.692′E, 07°00.075′N], and has a general thickness of about 12 m (Fig. 4B). At the base, the section consists of about 2.6 m thick greenish-yellow claystone, overlain by 1 m thick limestone, with clay inclusions at the base and visible quartz grains toward the top. This is overlain by 1.4 m thick moderately indurated limestone with about 90% of ooid-like fossils. Above this unit is about 4 m thick of dirty white limestone, with no visible fossil.

### Unit 2: Southwestern Mine (SWM)

This unit is exposed at the South-Western Mine (SWM) in the Ibese quarry (Fig. 5A), with coordinates [003°01.614′E, 06°59.440′N]. The sequence consists of limestone, glauconitic band, shale, and ferruginized sandstone, with a general thickness of approximately 23 m (Fig. 5B), dipping gently to the south. Below the sequence is about 5 m thick grey to dark, impure argillaceous limestone with abundant macrofossils. This is overlain by 3 m thick hydrated, dirty white limestone, with few macrofossils, followed by 2 m thick white limestone. Overlying this unit is 1 m thick green glauconitic band, which has a sharp contact with the underlying limestone unit. Above the glauconitic unit is about 10 m thick sequence of grey to dark, fissile shale, interbedded with approximately 1 m thick ferruginized sandstone unit. The fissile shale forms a sharp contact with the underlying glauconitic unit (Fig. 5A,B).

## Methods

The methods employed in the study included laboratory sample processing and transmitted light microscopic logging. Twelve (12) samples of limestone and shale were subjected to palynological sample processing for their palynomorph content (Table 1). The sample preparation was carried out, using the conventional method of acid maceration technique for recovering acid-insoluble organic-walled microfossils from sediments. Each sample was thoroughly cleaned to remove field contaminants. 10 g of each sample was weighed out in a standard weighing balance and gently crushed with agate mortal and piston. The crushed sample was digested for 30 minutes in 40% concentrated hydrochloric acid for removal of carbonate, and 72 hours in 48% concentrated hydrofluoric acid to remove silicates. The digested sample was diluted with distilled water and sieve-washed through 10 microns nylon mesh. The sieve-washed 10 g residues equivalent was partitioned into two parts, 5 g each, for oxidation and for kerogen assessment. The 5 g residue extract was oxidized for 30 minutes in 70% conc. HNO_3_ and 5 minutes in Schulze solution to render the fossils translucent for transmitted light microscopy. The acid-free oxidized residue was rinsed in 2% concentrated KOH solution to neutralize the acid; and swirled to remove the resistant coarse mineral particles and undigested organic matter. The swirled residue was collected on the sieve and stained with Safranin – O to increase the depth of contrast for microscopic examination and photography.

**Table 1:** The Occurrence and Distribution Chart of Palynomorphs’ Species in the examined samples.

Palynomorphs Species	NM03	NM04	NM05	NM06	SWM02	SWM03	SWM04	SWM06	SWM08
**TERRIGENOUS SPECIES**									
**Spores**									
*Cyathidites minor*	2	—	4	6	4	2	2	4	4
*Leiotriletes adriennis*	—	2	—	2	—	4	—	4	—
*Verrucatosporites usmensis*	2	4	—	—	4	2	1	—	—
*Laevigatosporites ovatus*	10	8	6	10	8	5	4	8	6
*Schizophacus sp*.	4	—	—	2	—	—	—	—	—
*Polypodiaceoisporites sp*.	—	2	—	—	2	4	—	—	—
**Pollen**									
*Proxapertites operculatus*	4	1	6	4	8	6	4	—	2
*Proxapertites cursus*	—	—	—	—	2	2	—	4	6
*Retibrevitricolpites triangulatus*	2	—	—	2	6	—	2	2	2
*Mauritidiites crassiexinus*	2	2	1	—	—	2	1	1	—
*Mauritidiites crassibaculatus*	2	—	—	—	2	—	—	—	—
*Grimsdalea polygonalis*	—	—	—	—	—	—	—	2	1
*Spinizonocolpites baculatus*	2	4	—	2	—	2	1	—	—
*Spinizonocolpites echinatus*	—	—	2	—	1	—	—	4	6
*Bombacidites sp*.	—	—	—	—	—	—	—	2	4
*Echitriporites trianguliformis*	2	4	—	2	—	4	1	—	—
*Scabratriporites simpliformis*	—	2	—	1	—	—	—	—	—
*Monoporites annulatus*	2	—	2	4	2	—	2	4	3
*Tricolpites hians*	3	2	1	—	—	2	1	3	1
*Striatopollis sp*.	1	—	—	—	—	—	—	4	2
*Psilatricolporites crassus*	5	—	4	1	3	—	6	—	—
*Retistephanocolpites williamsi*	—	—	—	—	—	2	—	2	4
*Retitricolporites irregulari*	—	—	—	—	3	—	—	4	2
*Psiltricolporites operculatus*	2	—	3	1	2	3	1	2	2
*Psilatriporites rotundus*	2	2	—	2	—	4	4	3	2
**MARINE SPECIES**									
**DinoFlagellate cysts**									
*Apectodinium homomorphum*	—	—	—	—	—	—	5	14	9
*Homotryblium abbreviatum*	—	—	—	—	—	—	2	4	8
*Apectodinium quinquelatum*	—	—	—	—	—	—	2	4	5
*Oligosphaeridium complex*	2	—	—	1	—	—	4	6	—
*Spiniferites ramosus*	—	—	—	—	—	—	2	—	4
*Glaphyrocysta ordinate*	—	1	2	—	2	4	—	—	2
*Adnatosphaeridium vitatum*	—	—	—	2	—	—	2	3	2
*Homotryblium pallidium*	—	—	—	—	—	—	1	3	4
*Kallosphaeridium yorubaensis*	—	—	3	1	—	—	—	6	4
*Cyclonephelium deckonincki*	—	—	—	—	—	—	—	2	—
*Diphyes colligerum*	2	1	—	—	—	2	—	4	6
*Operculodinium centrocarpum*	4	—	—	—	2	1	2	4	2
*Homotryblium tenuispinosum*	1	—	—	2	—	—	—	2	4
*Hystrichokolpoma sp*.	—	—	—	—	—	—	—	1	—
*Ifecysta sp*.	—	—	—	—	—	—	—	4	2
*Achomosphaera ramulifera*	—	—	—	—	—	—	—	—	2

The stained residue (aliquots) was dispersed with polyvinyl alcohol, dried on cover-slips and mounted in petro-poxy resin. One slide was made from each sample, scanned and logged under the transmitted light microscope. Light photomicrographs were taken with a Leica III binocular microscope.

## Results

**Figure 6:**
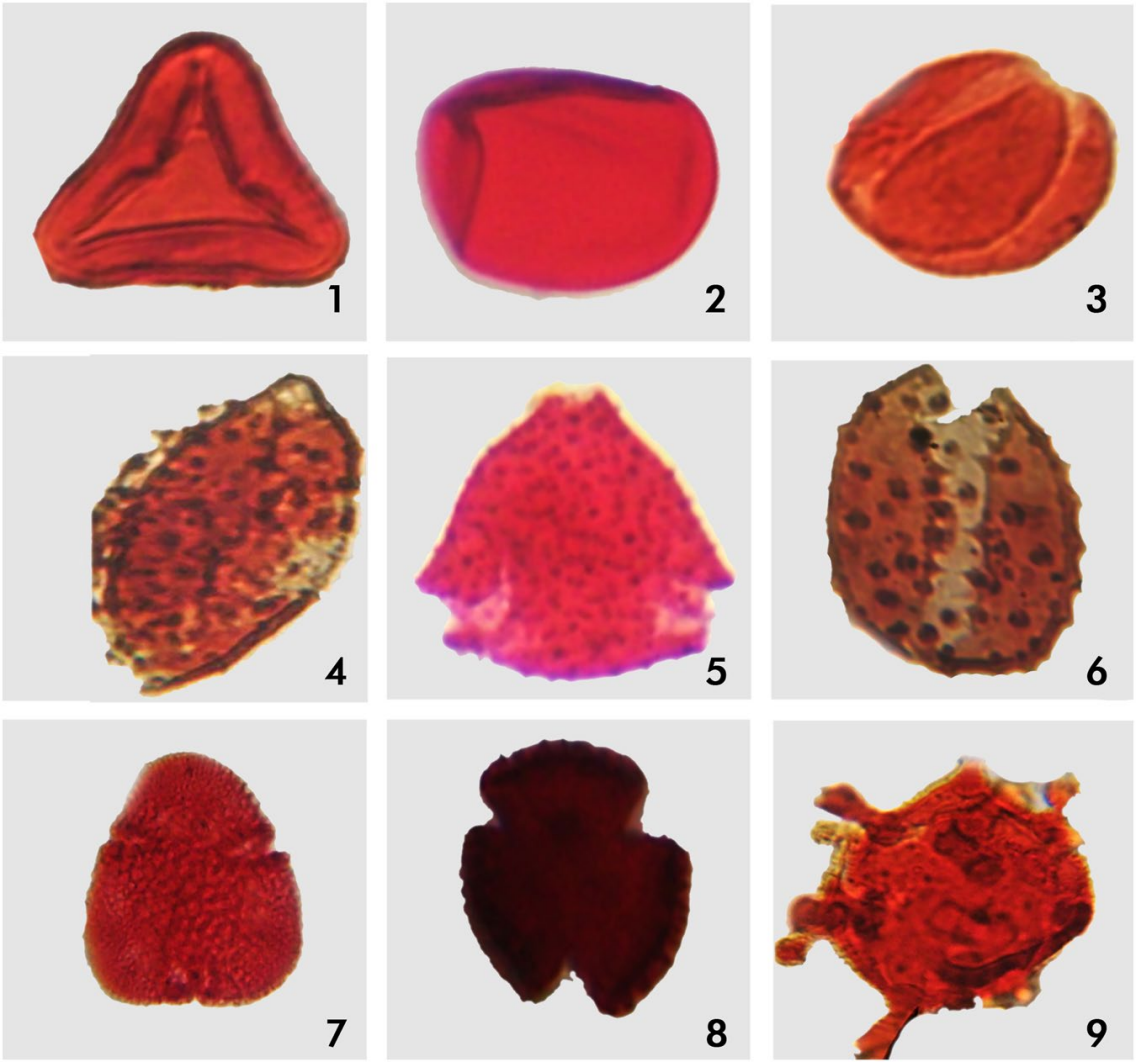
Micrographs of some palynomorphs recovered from the analyzed samples. Magnifications nos. 5, 7 and 9 (X 100 oil immersion), others (X 40). 1. Cyathidites minor. 2. Laevigatosporites ovatus. 3. Proxapertites operculatus. 4. Mauritidiites crassibaculatus. 5. Echitriporites trianguliformis. 6. Spinizonocolpites baculatus. 7. Bombacidites annae. 8. Retibrevitricolpites triangulatus. 9. Grimsdalea polygonalis.

**Figure 7:**
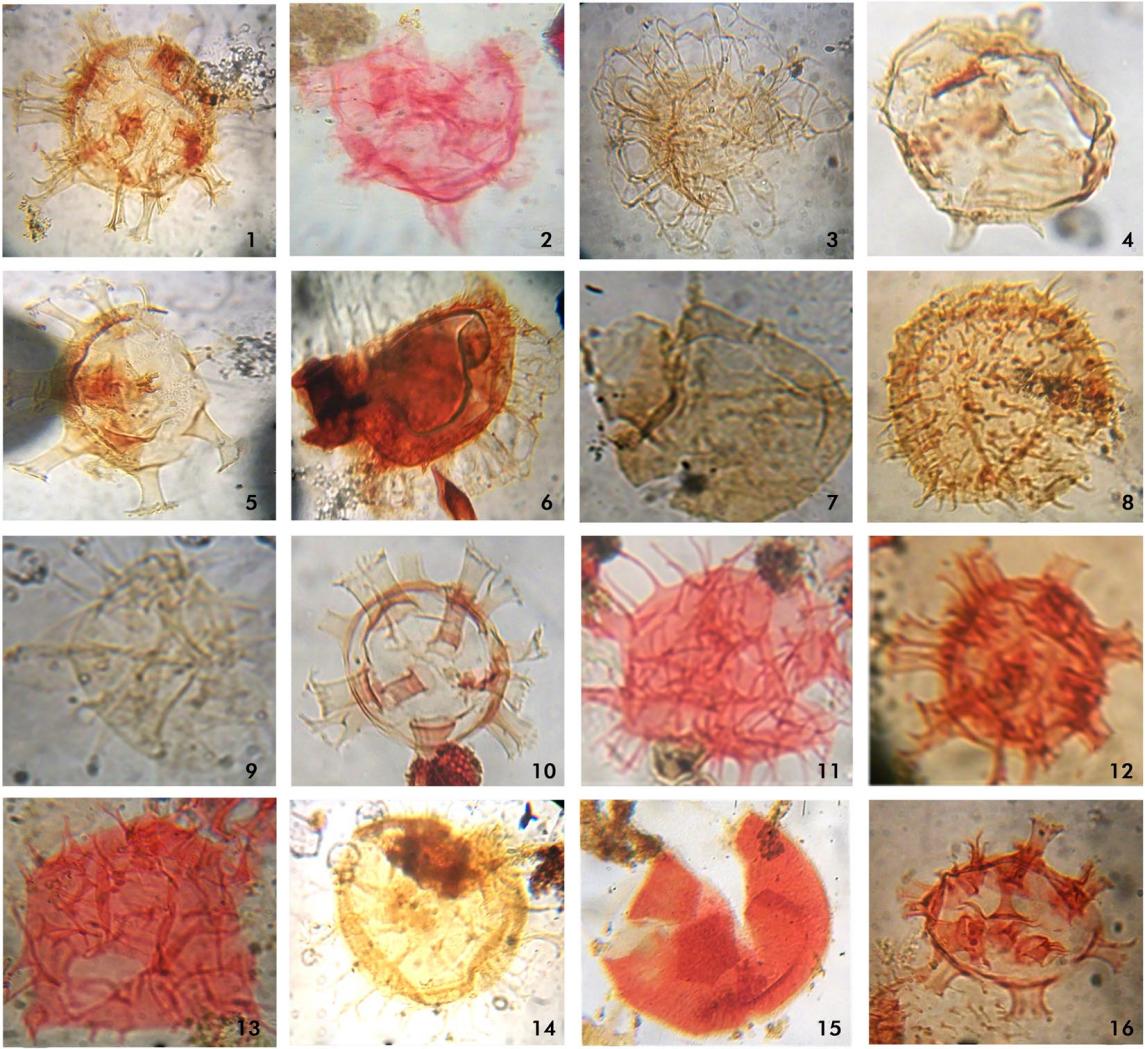
Micrographs of some palynomorphs recovered from the analyzed samples. Magnifications nos. 12, 14, 15 and 16 (X100 oil immersion), others (X 40). 1 Oligosphaeridium complex, 2. Homotryblium pallidium. 3. Adnatosphaeridium vitatum. 4. Ifecysta sp. 5. Hystrichokolpoma sp. 6. Glaphyrocysta ordinata. 7. Ifecysta sp. 8. Operculodinium centrocarpum. 9. Diphyes colligerum. 10. Homotriblium tenuispinosum. 11. Apectodinium homomorphum. 12. Oligosphaeridium complex. 13. Apectodinium quinquelatium. 14. Spiniferites ramosus. 15. Kallosphaeridium yorubaensis. 16. Homotryblium abbreviatium.

**Figure 8:**
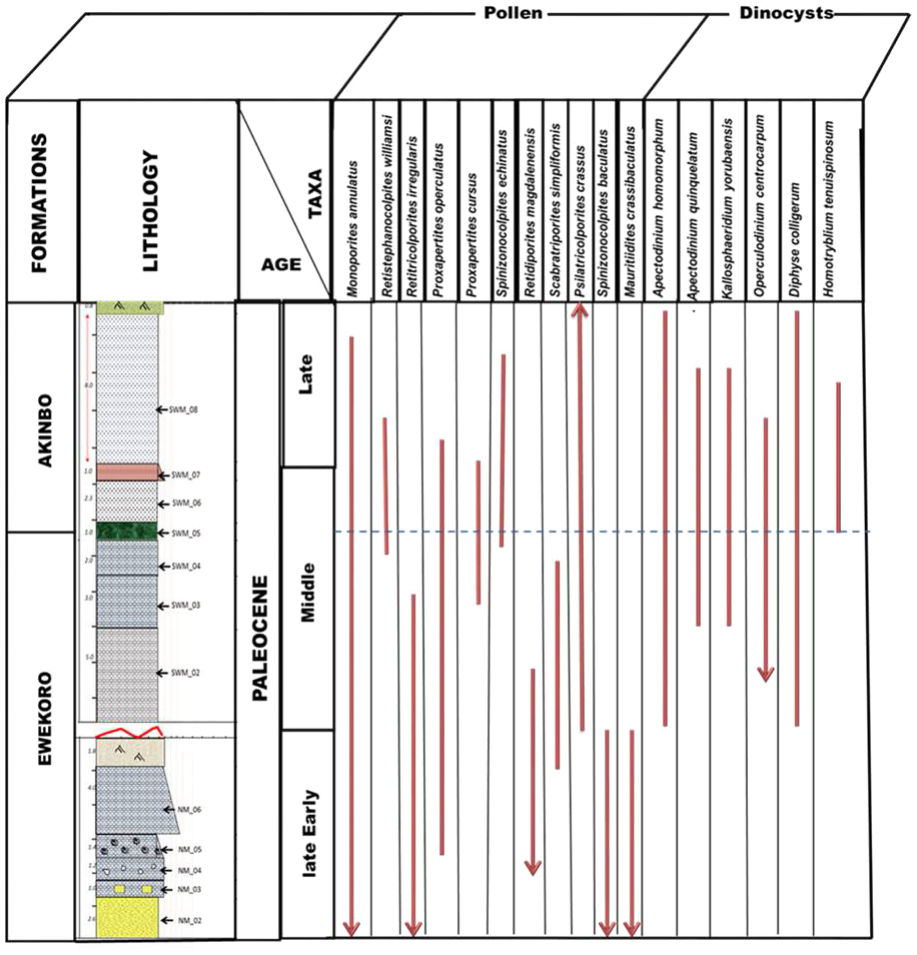
Stratigraphic range chart of some key palynomorphs recovered.

Table 1, shows the absolute occurrence and distribution of palymomorph counts present in the examined samples from the Units 1 and 2. The carbonaceous fissile shale samples recorded moderately rich palynomorph assemblage while the white indurated limestone samples yielded moderate to low palynomorph counts. The claystone (NM02), argillaceous limestone (SWM02), glauconite (SWM 05), and ferruginized sandstone (SWM07) samples were barren of microfossils (Table 2). The white indurated limestone samples recorded more terrigenous (spores and pollen) species than the marine dinoflagellate cysts. Among the terrigenous species, pollen was the most abundant and diverse. The fissile shale samples also produced more terrigenous species (52–53%) than the marine microplanktons (48–47%). The pollens (39–41%) predominate over spores (12–13%) and were also more abundant and diverse (Fig. 8). The freshwater algal spores yielded very low count. Among the marine dinoflagellate species, the peridiniacean species, with proximate cyst affinity, were more abundant and diverse than the gonyaulacaceans species (Table 1) (Figs. 6 and 7).

**Table 2:** Frequency Distribution of the Palynomorphs (%) and their Paleoenvironmental Inferences.

SAMPLE NO.	PALYNOMORPHS (%) FREQUENCY			PALEO-SALINITY	PALEOENVIRONMENTS OF DEPOSITION
	Spores	Pollen	Marine Species		
	13%	39%	48%	Brackish water	Marginal marine (estuary)
SWM07	0%	0%	0%	Non-diagnostic	Barren
SWM06	12%	41%	47%	Brackish water	Marginal marine (estuary)
SWM05	0%	0%	0%	Non-diagnostic	Barren
SWM04	20%	65%	15%	Normal marine	Shallow marine
SWM03	37%	53%	10%	Normal marine	Shallow marine
SWM02	36%	60%	4%	Brackish water	Marginal marine (Proximal estuary)
NM06	36%	43%	21%	Normal marine	Shallow marine
NM05	29%	59%	12%	Normal marine	Shallow marine
NM04	36%	55%	9%	Normal marine	Shallow marine
NM03	35%	50%	15%	Normal marine	Shallow marine
NM02	0%	0%	0%	Non-diagnostic	Barren

## Discussion

### Age determination/correlation

Figure 8 shows the stratigraphic range chart of selected key palynomorph species recovered in the studied sections. The limestone samples from the Ewekoro Formation at Units 1 and 2 were dated **late Early Paleocene to early Middle Paleocene**, based on the presence of the following pollen key-taxa: *Proxapertites operculatus, Retidiporites magdalenensis, Spinizonocolpites baculatus, Mauritidiites crassiexinus, Scabratriporites simpliformis*, and *Echitriporites trianguliformis*, while the carbonaceous shales from the overlying Akinbo Formation were assigned **late Middle Paleocene to late Paleocene**, based on the following index-taxa: *Proxapertites operculatus /cursus, Grimsdalea polygonalis, Retibrevitricolpites triangulatus, Psilatricolporites operculatus, Scabratriporites simpliformis Retistephanocolpites williamsi, Bombacidites sp*.^23–28^ (Fig. 8). The age is strengthened by the occurrence of the well-known stratigraphically significant dinoflagellate cysts association, *Apectodinium homomorphum* (in overwhelming abundance), *Homotryblium* spp*., Apectodinium quinquelatum, A. abbreviatum, Operculodinium centrocarpum, Ifecysta* spp*., Cyclonephelium deckonincki, Diphyes colligerum, Kallosphaeridium yorubaensis, Adnatosphaeridium* sp and *Hystrichokolpoma* sp.^23,25^. Moreover, the total absence of typical Earliest Paleocene (Danian) marine dinoflagellates cyst association such as *Damassadinium californicum, Eisenack circumtabulata*, *Carpatela spp, Senoniasphaera* sp. and *Trichodinium* sp., in the examined limestone samples at the base of the Ewekoro Formation eliminates ages younger than **late Early Paleocene** for the formation.

The above recovered sporomorph assemblage from the Ewekoro Formation correlates with the base of the **pantropical** *Proxapertites operculatus* Zone of^27^, based on the presence of the following pollen key-taxa: *Proxapertites operculatus, Retidiporites magdalenensis, Spinizonocolpites baculatus*, and *Echitriporites trianguliformis*. On the basis of Niger Delta pollen zonations, the top of the Ewekoro Formation (early Middle Paleocene) correlates with the base of **P200 (**Middle Paleocene) pollen zone of^29^, and with the Middle Paleocene miospore (**Zone E)** of^30^ in the Gbekebo-l well, Benin Flank, southwestern Nigeria.

The sporomorph assemblage recovered from the Akinbo Formation (**late Middle Paleocene to Late Paleocene)** corresponds with the middle – late Paleocene **pantropical** *Proxapertites operculatus* Zone of^27^, due to the presence of the following pollen key-taxa: *Proxapertites operculatus, Proxapertites cursus, Spinizonocolpites echinatus, Retistephanocolpites williamsi* and *Retibrevitricolpites triangulatus*. The assemblage correlates with the middle – late Paleocene pollen zone (**P200 to P300)** of^29^, and with the middle – late Paleocene palynological assemblage **(Zone A)** of^28^, in the Niger Delta Basin, based on the presence of *Scabratriporites simpliformis, Proxapertites operculatus, Retibrevitricolpites triangulatus, Proxapertites cursus, Spinizonocolpites echinatus, Echitriporites trianguliformis, Bombacidites* sp. and *Grimsdalea polygonalis*, (Fig. 8). The formation further correlates well with the middle-late Paleocene miospore (**Zone E – G)** of^24^ in Gbekebo-l well, Benin Flank, southwestern Nigeria, based on the presence of the following key-taxa *Proxapertites cursus*, *Verrucatosporites usmensis* and *Spinizonocolpites echinatus*, and also coincides with the Paleocene (Selandian – Thanetian) dinoflagellate cysts (**Zone 2 – 4)** of^31^ based on *Apectodinium* spp*., Ifecysta* spp*., Homotryblium abbreviatum*, and *Kallosphaeridium yorubaensis*.

### Paleoenvironments of deposition

Table 2 shows the summary of palynomorphs percentage frequency distributions and their paleoenvironmental inferences. The environmentally significant palynomorphs encountered in the examined samples include *Proxapertites, Spinizonocolpites*, *Mauritiidites, Laevigatosporites, Verrucatosporites usmensis*, and *Polypodeaceoisporites*. *Proxapertites, Spinizonocolpites*, *Mauritiidites*, and *Longapertites* are pollen of palmae inhabiting similar environment as mangrove swamp of the humid tropics^32–38^.

*Laevigatosporites, Verrucatosporites usmensis* and *Polypodeaceoisporites* are fern spores of swampy fresh water^27,39^. The presence of these species together with the co-occurrence of the monocolpate pollen *Proxapertites, Spinizonocolpites*, and *Mauritiidites*, and minor influx of the dinoflagellates species (marine influence), in the samples (NM03, NM04, NM05, NM06, SWM03, SWM04, and SWM06), from the Ewekoro Formation, indicate possible deposition in a coastal/shallow marine environment in the lower deltaic settings (Table 2).

Moreover, there is a mixed occurrence of freshwater (non-brackish) species in different proportions, with high dominance of nearshore brackish water forms in the carbonaceous shale samples (SWM06 and SWM08), from the Akinbo Formation. The presence of *Verrucatosporites usmensis, Cyathidites* spp. and *Laevigatosporites* spp., together with dinoflagellates cysts association*, Apectodinium homomorphum*, *A. quinquelatum, A. abbreviatum, Kallosphaeridium sp*. and *Homotryblium sp.,* in the samples, show strong indication of deposition in a marginal marine/or nearshore brackish water-estuarine environment, within the lower deltaic setting (Table 2).

## Summary and Conclusion

Palynological study of the Paleogene succession exposed at the Ibese quarry, within the Eastern Dahomey (Benin) Basin has been undertaken. Two formations, the Ewekoro and Akinbo, were identified in the area of study. The result from the palynological analysis of collected field samples revealed a **late Early Paleocene – early Middle Paleocene** age for the Ewekoro Formation, and **late Middle Paleocene – late Paleocene** for the Akinbo Formation. Palynomorphs of environmental value indicated differences in paleodepositional environments, ranging from shallow marine in the Ewekoro Formation at the base, up the stratigraphic sequence, to marginal marine estuarine depositional environment in the overlying Akinbo Formation.

The new age designations are correlated to other existing age/biozones in the subsurface series of the Niger Delta Basin, and with the pantropical palynological zones of^27^ and the palynofloral provinces of^40^, in the tropical areas of Africa and northern South America. Thus, moreover, comparing the designated age strata in southwestern Nigeria with the international palynozones worldwide will be meaningful for future works.

The results presented in this work provide a better insight into the palynological biostratigraphy of the basin. Further studies, especially in the aspects of micropaleontology and nanofossils biostratigraphy, are required to strengthen the age designation and paleoenvironmental study for the strata in the area. Furthermore, it becomes necessary to add new results to the existing database as further studies are carried out in order to integrate results from this study with subsequent ones, thereby providing adequate basin-wide, regional correlation.
